# Non-vitamin K Antagonist Oral Anticoagulants and Drug-Food Interactions: Implications for Clinical Practice and Potential Role of Probiotics and Prebiotics

**DOI:** 10.3389/fcvm.2021.787235

**Published:** 2022-01-17

**Authors:** Ana Sánchez-Fuentes, José Miguel Rivera-Caravaca, Raquel López-Gálvez, Francisco Marín, Vanessa Roldán

**Affiliations:** ^1^Department of Hematology and Clinical Oncology, Instituto Murciano de Investigación Biosanitaria (IMIB-Arrixaca), Hospital General Universitario Morales Meseguer, University of Murcia, Murcia, Spain; ^2^Department of Cardiology, Instituto Murciano de Investigación Biosanitaria Arrixaca, Centro de Investigación Biomédica en Red - Enfermedades Cardiovasculares, Hospital Clínico Universitario Virgen de la Arrixaca, University of Murcia, Murcia, Spain; ^3^Liverpool Centre for Cardiovascular Science, Liverpool Heart and Chest Hospital, University of Liverpool, Liverpool, United Kingdom

**Keywords:** non-vitamin K antagonist oral anticoagulants (NOACs), drug interactions, food interactions, P-glycoprotein, cytochrome P450 (CYP) 3A4, probiotics

## Abstract

Non-vitamin K antagonist oral anticoagulants (NOACs) are a therapeutic option to prevent stroke in patients with atrial fibrillation (AF). In fact, NOACs have become the recommended choice by international clinical practice guidelines over vitamin K antagonists (VKA), because of their efficacy and safety profile, especially in newly initiated patients. The more predictable pharmacokinetic and pharmacodynamic profile of this family of drugs allows preventing anticoagulation drug monitoring. Furthermore, NOACs have significantly fewer drug and food interactions in comparison with VKAs. Despite this, there are no studies that compare the effects on the quality of anticoagulation of NOACs with the intake of potential interactions drugs of P-glycoprotein and cytochrome P450 (CYP). This review brings an overview of NOACs pharmacokinetics profile and their potential drug-food interactions. We also briefly discuss the potential role of prebiotics and probiotics in patients under therapy with NOACs.

## Introduction

Non-vitamin K antagonist oral anticoagulants (NOACs), including rivaroxaban, apixaban and edoxaban as factor Xa inhibitors, and dabigatran etexilate as a thrombin inhibitor, are the first-line treatment for most patients with venous thromboembolism (VTE) and for stroke prevention in atrial fibrillation (AF) (1–3).

It is well known that NOACs have a lower propensity for drug and food interactions compared to vitamin K antagonists (VKAs), due to their individual coagulation proteins target and to the administration in fixed-dose without requiring routine coagulation monitoring (4). Even though, variations in NOACs concentration may occur with the concomitant use of inducers or inhibitors of P-glycoprotein (P-gp) and cytochrome P450 (CYP) 3A4, thus increasing thrombotic and bleeding risk, respectively (5–8).

To date, there is scarce evidence about the potential interactions of herbal products, dietary supplements, and food with NOACs. Moreover, the use of probiotics and prebiotics is currently increasing due to their multiple benefits described in the human body, but it is unclear whether probiotics and prebiotics might have a potential influence on NOACs pharmacokinetic parameters (9). The aim of this mini-review article is to summarize the present knowledge on potential drug-food/herbal products interactions of NOACs and the potential implications of increased use of products such as probiotics and prebiotics.

### Brief Summary of the NOACs Pharmacology

A summary of the different stages of NOACs pharmacology is shown in Table 1.

**Table 1:** Pharmacological properties of non-vitamin K antagonist oral anticoagulants (NOACs).

	**Dabigatran**	**Apixaban**	**Edoxaban**	**Rivaroxaban**
**Target**	Thrombin	Factor Xa	Factor Xa	Factor Xa
**Bioavailability, %**	3–7%	50%	62%	15 mg/20 mg: 66% without food, 80–100% with food
**Plasma protein binding**	35%	87%	55%	95%
**Prodrug**	Yes	No	No	No
**Clearance non-renal/renal of absorbed dose**	20%/80%	73%/27%	50%/50%	65%/35%
**Dialysability**	50–60% (in part dialysable)	14% (not dialysable)	NA (not dialysable)	NA (not dialysable)
**Time to peak levels (h)**	3	3	2–4	2–4
**Elimination half-life (h)**	12–17	12	10–14	5–9 (young) 11–13 (elderly)
**Metabolism**	Glucuronic acid conjugation	CYP3A4 (25%), CYP1A2, CYP2J2, CYP2C8, CYP2C9, CYP2C19	CYP3A4 (<4% of elimination)	CYP2A4 (18%), CYP2J2
**Absorption with food**	No effect	No effect	6–22% more; minimal effect on exposure	+39% more
**Absorption with H2B/PPI**	−12% to 30% (not clinically relevant)	No effect	No effect	No effect
**Drug interactions**	Inhibitors and inducers of P-gp	Dual inhibitors and inducers of CYP3A4 and P-gp	Inhibitors and inducers of P-gp	Dual inhibitors and inducers of CYP3A4 and P-gp

*P-gp, P-glycoprotein; NA, not applicable; H2B, H2 receptor blockers; PPI, proton pump inhibitor*.

*This table is based on evidences from Stangier et al., Raghavan et al., Ogata et al., Kubitza et al. and Mueck et al. (46–50)*.

VKAs have several interactions with foods and herbal supplements (10, 11). These interactions can significantly increase the risk of thromboembolic and bleeding events due to phenomena of over or under anticoagulation (8, 11). The introduction of NOACs has partly solved this problem. Despite this family of drugs presents fewer drug-food interactions compared to VKAs, they are not free of potential interactions, and therefore some international guidelines suggest considerations with some herbal medicines or food that can affect plasma levels of NOACs (12).

Metabolism and elimination pathways of NOACs depend on the intestinal transporter P-gp (for dabigatran, apixaban, edoxaban and rivaroxaban) and the CYP3A4-type cytochrome P450 (for apixaban and rivaroxaban) (13, 14).

Firstly, P-gp transporters are efflux carriers that can be found in different epithelia of the human body, highlighting their presence and function in the luminal membrane of the small intestine, proximal renal tubules or hepatocytes (15). P-gp is essential for gastrointestinal resecretion after intestinal absorption, and it is also actively involved in the renal secretion of NOACs (12, 16). Because P-gp has high transport capacity but low specificity for different substrates, competitive inhibition or induction of the P-gp pathway will affect plasma concentration of those drugs that share the same metabolic pathway (15, 17).

**Figure 1:**
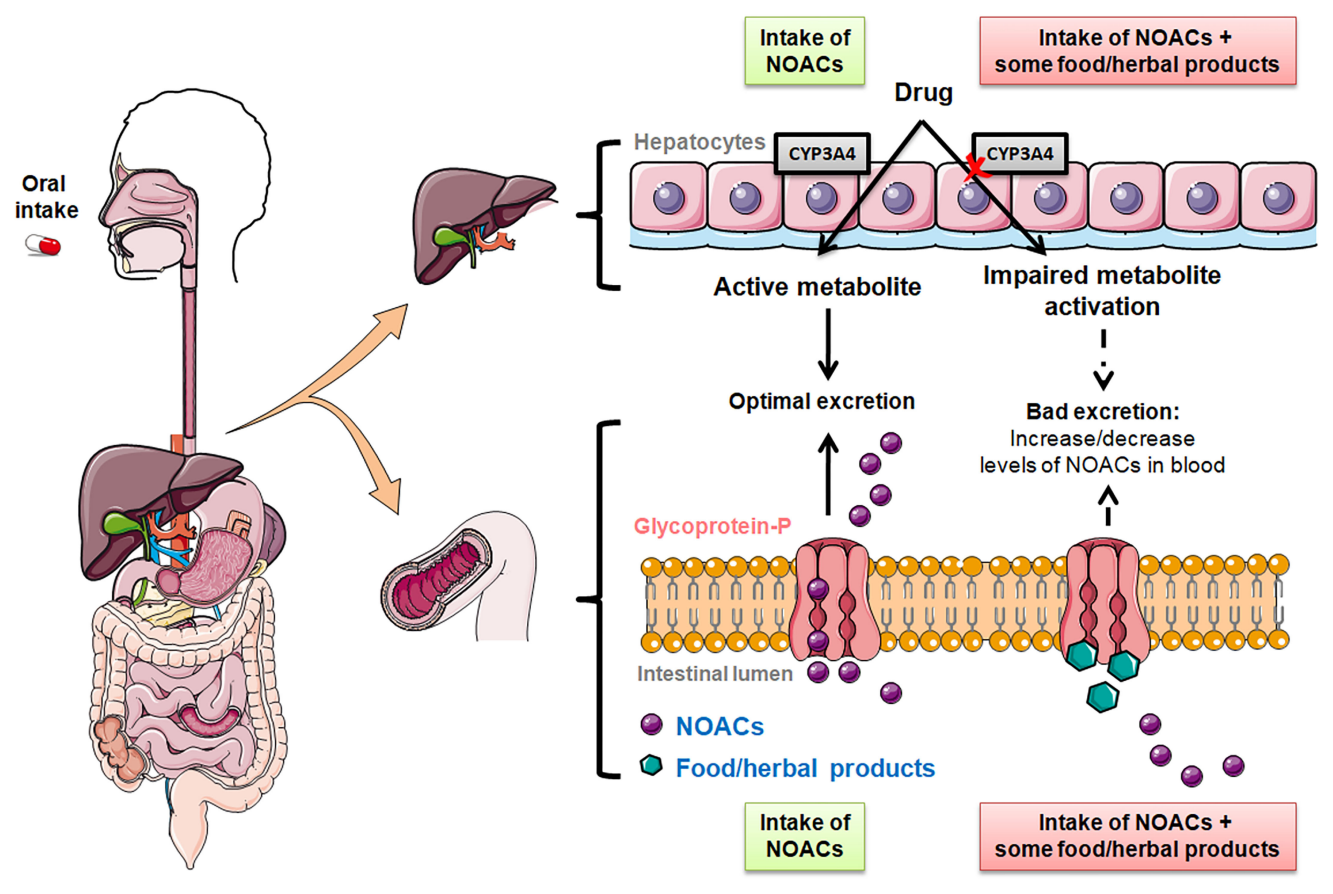
Effect of the intake of food/herbal products on the metabolization of NOACs. Elimination of NOAC mediated by P-glycoprotein and cytochrome P450 (CYP) 3A4 in the intestinal lumen and liver.

Secondly, cytochrome P450, specifically isoenzyme CYP3A4, is essential for the hepatic elimination of some NOACs (apixaban and rivaroxaban) (16). Regarding clearance of other foreign substances, most of them depend on CYP1, 2, and 3 families, although up to 57 functional CYPs have been described in the human body (18). As in case of P-gp, strong inhibition or induction of CYP3A4 can affect plasma levels of NOACs (19). Thus, modifications in these metabolic pathways in the presence of certain foods and drugs could influence plasma NOACs concentrations and the safety of treated patients if the therapeutic objective is not reached (13, 14). Therefore, strong inducers or inhibitors of P-gp and CYP3A4 should be used carefully or even avoided with the concomitant intake of NOACs (20) (Figure 1).

### Effect of Food and Herbal Products on the NOACs Pharmacology

Although food and herbal inducers or inhibitors of P-gp or CYP3A4 are supposed to interfere with the pharmacokinetics of NOACs (see Table 2), no direct association of these interactions is available in humans and *in vivo* studies (5). Limited evidence showed that rivaroxaban bioavailability improves when the drug is taken with food (21, 22), so that it is recommended to take it with food. However, food intake does not influence the bioavailability of the other NOACs.

**Table 2:** Foods and herbal drugs that modulate P-gp and CYP3A4 activity (8, 25, 26).

	**P-glycoprotein**	**CYP3A4**
Inhibitors	Humans: *Ginkgo biloba, berberine*. Animal models: *Black pepper, grape juice, apigenin, rutin, capsaicin*. *In vitro*: *Lemonin, soybean extract, notoginsenoside R1, curcumin, green tea, fisetin, honokiol, ginger*.	Echinacea purpurea (*). Garlic (*). Valerian (*). *Plants from Traditional Chinese Medicine (*Acacia catechu, Andrographis paniculata, Arctium lappa, Areca catechu, Bupleurum marginatum, Dysosma versipellis*, and S*patholobus suberectus). *In vitro*: Saw palmetto (Serenoa repens)
Inducers	Humans*: St. John's wort*. Animal models: *Quercetin, scutellaria, soy milk and miso, sucralose, licorice root*. *In vitro*: *Genipin, mango*.	*St. John's wort*.

(*) *Mild CYP3A4 inhibition*.

*This table is based on evidences from Di Minno et al., Mousa et al., Tsai et al., Ashour et al., Yale et al. and Buhner SH (13, 28, 29, 31, 51, 52)*.

On the other hand, although there is no evidence from clinical studies, it has been proposed that grapefruit could influence the bioavailability of rivaroxaban (12, 13, 21–26). Another example is St. John's wort, a known potent inducer of P-gp and CYP3A4. Therefore, it is expected that NOACs, as substrates of P-gp (all of them) and CYP3A4 (rivaroxaban and apixaban), have lower plasma levels with the concomitant intake of St. John's wort, which potentially increases stroke risk (13). Thus, co-administration of this herbalist product with apixaban and edoxaban should be made with caution, and even avoided with rivaroxaban and dabigatran (13, 27). On the contrary, some herbal medicines and foods have antiplatelet and anticoagulation effects, such as garlic, ginger, ginkgo Biloba, ginseng, green tea, or horse chestnut (13, 28, 29). Nowadays, there is not enough information available to confirm possible interactions between these herbal products/foods and NOACs. Even so, as a preventive measure, caution should be exercised in patients with risk factors for bleeding or polypharmacy (17).

But importantly, it should be noted that several food and herbal products with potential interactions with NOACs are common and easily accessible. A recent study involving patients taking apixaban demonstrated that the prevalence of use of over-the-counter products (i.e., Chinese herbs [including products such as danshen, dong quai, and ginseng], ginger, ginkgo biloba, herbal teas [including green and chamomile tea], St. John's wort, and turmeric) with potentially serious apixaban interactions, was high. The most common dietary supplements with potential apixaban interactions of increased bleeding were herbal teas and turmeric. Of note, less knowledge about over-the-counter products with potentially serious interactions was associated with greater use of these products (OR 0.54; 95% CI 0.35–0.85) (30). However, this study did not report association regarding potential adverse events with concomitant use of over-the-counter products and apixaban. Such results could be relevant since *in vitro* studies have demonstrated that widely used plants from Traditional Chinese Medicine may inhibit CYP3A4, some of them in a proportion higher than 85% when used at a dose of at least 100 μg/mL (31).

Notwithstanding this is a field still to be explored that requires further studies. Indeed, the available evidence is currently so limited that it is not possible to give firm advices. In this regard, some previous manuscripts -mostly case studies, have hypothesized the potential interaction of herbal products and NOACs. A case study reported an isolated hemopericardium in a patient with AF taking rivaroxaban. The authors suggested that a possible contributor to hemopericardium could be the concomitant use of saw palmetto, an herbal medication from the extract of the berries of *Serenoa repens*/*Serenoa serrulata* commonly used for symptom relief in benign prostate hyperplasia, which may inhibit CYP3A4 activity (32). More recently, a report introduced a case of a patient presenting severe (and fatal) gastrointestinal bleeding after the self-administration of a boiled mixture of ginger and cinnamon twice daily for 3 days in concomitant use with dabigatran. Ginger is a P-gp inhibitor, which may increase dabigatran concentration, whereas Cassia cinnamon (the most common one) is considered a rich source of coumarin. Thus, Ginger and cinnamon taken together with dabigatran, in this case, might increase dabigatran levels and also add a second oral anticoagulant (coumarin) to dabigatran, thus significantly increasing the risk of bleeding (33). In the same line, Gressenberger and collaborators published a case of a young male patient under rivaroxaban due to recurrent spontaneous deep vein thrombosis and a heterozygous Factor-V-Leiden mutation which presented sudden onset of hemoptysis. The patient was not taking any other concomitant medication which may justify the bleeding, but reported a constant intake of three liters of home-brewn ginger tea per day during the last month (34).

In summary, strong P-gp and CYP3A4 inducers and inhibitors should be avoided concomitant to NOACs. In the case of mild potential pharmacodynamic interactions, special care is needed particularly in high-risk patient groups. Patients who develop bleeding or thromboembolic events during NOAC therapy should be investigated for co-medications, intake of herbal drugs, and dietary habits (13). Pharmacokinetic interactions of accompanying drugs need to be considered when prescribing NOACs.

### NOACs and Gut Microbiota: Potential Role of Probiotics and Prebiotics

Gut microbiota is shaped by a large ecosystem of microorganisms that live or move through the skin, genitourinary, gastrointestinal and respiratory tracts (35). It has been related to very relevant roles in the human body, highlighting its benefits in immunological, nutritional and metabolic processes capable of influencing pharmacological reactions and even in the safety of drugs (35, 36).

However, the interaction of this complex system with oral anticoagulants has not yet been fully clarified. In the case of VKAs, the role of intestinal bacteria in vitamin K production has been suggested. For example, there are situations in which the population of the bacterial flora of the gut is reduced, causing a decrease in the source of vitamin K production, as in the case of the use of antibiotic therapies. Due to the decrease in vitamin synthesis, the anticoagulant effect may increase, as well as its possible adverse clinical effects (37, 38).

Otherwise, part of the metabolic capacity of gut microbiota is suggested to be closely associated with the presence of the cytochromes, especially CYP3A4-type cytochrome P450, which is highly expressed in the mucosa of the small intestine (38). Besides, the concomitant function of P-gp and CYP3A4 is particularly important in the gut, which could restrict oral drug bioavailability of a wide variety of substrates, without a clear known implication with NOACs to date (39).

Nowadays there is a growing interest in the use of probiotics to modify the composition and roles of the gut microbiota. Probiotics are defined as live microorganisms that, in optimal proportions, provide beneficial effects on the host and it is already known that they can have an impact on the gut ecosystem (9, 39–41). Among these actions, it has been observed that probiotics can interact with host cells through chemical signals and produce short-chain-fatty acids as metabolic products capable of influencing commensal microbes, or on the contrary, against potential pathogens as immune mechanisms (42). The species most commonly used as probiotics are *Lactobacillus* and *Bifidobacterium*, followed by the yeast *Saccharomyces boulardii* and some *E. Coli* and *Bacillus* species (42). In the particular context of oral anticoagulants, a recent *in vitro* study showed a significant change in the concentration of acenocoumarol (a VKA) after incubation with bifidobacteria, results which are compatible with biomodification of the drug due to enzymatic activity of bifidobacteria (43). However there are no data regarding NOACs.

On the other hand, prebiotics are selectively fermented ingredients, most of them used as food ingredients like cereals, spreads, or biscuits for example (42, 44). Inclusion in these products is shaped like lactulose or breast milk oligosaccharides, oligofructose among others (40). As with probiotics, prebiotics also influence the activity and composition of the gut microbiota (44). Intake of prebiotics decreases potential pathogens and increases the number of beneficial anaerobic bacteria (42).

In addition, changes in the bacterial population, also called dysbiosis, and its metabolic products have been related to pathogenetic mechanisms of many infections, inflammatory diseases and cardiovascular diseases (45). All this evidence is increasing the interest on the use of probiotics and prebiotics, given the benefits observed in improving the antigenic immune response, regulating intestinal inflammation and bolstering the intestinal barrier (41). In some populations at risk, it could be relevant to encourage probiotic and prebiotics research to deepen knowledge of the influence of changes in bacterial activity and its composition, and how this might relate with the intake of NOACs, but this evidence is far from complete (35).

## Conclusions

There is scarce evidence so far regarding the interaction of NOACs with food and herbal products. However, some herbals and food modulate P-gp and CYP3A4 activity, so their potential impact on the anticoagulant effect of NOACs should be carefully examined. Thus, in order to improve the safety profile of NOACs with potential interactions, more clinical studies are needed to provide information from real-world data.

Finally, novel therapies such as probiotics/prebiotics might confer health benefits by modifying gut microbiota composition and its activity, but the effect on the safety and efficacy of NOACs is still unclear.

## Author Contributions

AS-F, JMR-C, and RL-G: drafted the manuscript. RL-G: designed and performed the figure. FM and VR: conceptualized the study, supervised the study, and made a critical revision of the manuscript. All authors listed have made a substantial, direct, and intellectual contribution to the work and approved it for publication.

## Funding

JMR-C has received a grant from the Sociedad Española de Trombosis y Hemostasia (grant for short international training stays 2020) and the First Contact Initiative Grant 2020 from the European Society of Cardiology Council on Basic Cardiovascular Science. This work was supported by the Spanish Ministry of Economy, Industry, and Competitiveness, through the Instituto de Salud Carlos III after independent peer review (research grant: PI17/01375 co-financed by the European Regional Development Fund) and group CB16/11/00385 from CIBERCV.

## Conflict of Interest

The authors declare that the research was conducted in the absence of any commercial or financial relationships that could be construed as a potential conflict of interest.

## Publisher's Note

All claims expressed in this article are solely those of the authors and do not necessarily represent those of their affiliated organizations, or those of the publisher, the editors and the reviewers. Any product that may be evaluated in this article, or claim that may be made by its manufacturer, is not guaranteed or endorsed by the publisher.
